# Three-Dimensional Assessment of Volumetric Changes in Sinuses Augmented with Two Different Bone Substitutes

**DOI:** 10.1155/2016/4085079

**Published:** 2016-07-19

**Authors:** B. Alper Gultekin, Oguz Borahan, Ali Sirali, Z. Cuneyt Karabuda, Eitan Mijiritsky

**Affiliations:** ^1^Department of Oral Implantology, Istanbul University Faculty of Dentistry, Istanbul, Turkey; ^2^Department of Oral and Maxillofacial Radiology, Marmara University Faculty of Dentistry, Istanbul, Turkey; ^3^Department of Periodontology, Bezmialem Vakif University Faculty of Dentistry, Istanbul, Turkey; ^4^Department of Oral Rehabilitation, Maurice and Gabriela Goldschleger School of Dental Medicine, Tel-Aviv University, Tel Aviv-Yafo, Israel

## Abstract

*Introduction*. The bone volume of the posterior maxilla may not be appropriate for implant placement, due to factors such as pneumatized maxillary sinus. The purpose of this study was to evaluate the percentage of graft volume reduction following sinus floor elevation (SFE), with either slow resorbable bone substitute only or a composite of slow and fast resorbable bone substitutes, using cone beam computed tomography (CBCT).* Materials and Methods*. In this retrospective study, CBCT scans of SFE procedures were evaluated to determine the volume of grafted sinus with either deproteinized bovine bone (DBB) or a 2 : 1 mixture of biphasic calcium sulfate (CS) and DBB, as a composite. The volumetric changes of sinus augmentations were measured 2 weeks (V-I) and 6 months (V-II) after operation.* Results*. Thirty-three patients were included in this study. The average percentage volume reduction was 9.39 ± 3.01% and 17.65 ± 4.15% for DBB and composite grafts, respectively. A significant graft volume reduction was observed between V-I and V-II for both groups (*p* < 0.01). The DBB group exhibited significantly less volume reduction than the composite group (*p* < 0.01).* Conclusions*. Augmented sinus volume may change before implant placement. DBB offers greater volume stability during healing than composite grafts.

## 1. Introduction

Implant-supported restorations have become a successful and predictable choice of treatment for patients who have sufficient bone volume available [1]. After tooth loss, maxillary sinus enlargement and resorption of the alveolar ridge may be observed in the edentulous posterior maxilla [2]. Sinus floor elevation (SFE) has been accepted as a common treatment method in cases of atrophy of the posterior maxilla, as it has high predictability and low intra- or postsurgical complication rates [3].

Although SFE has become a standard treatment procedure since the early 1980s, there is currently no evidence supporting an ideal graft material for this approach [3]. Various grafting materials have been used for SFE, including autogenous bone, allogenic grafts, xenogenic grafts, alloplasts, and a mixture of these materials as a composite [4]. The ideal bone replacement biomaterial for SFE should be biocompatible, resorbed quickly, replaceable by newly formed bone, and able to create adequate volume for implant stability [5]. Autogenous bone grafts are considered as the gold standard in augmentation procedures because of their unique osteogenic, osteoinductive, and osteoconductive properties [5, 6]. However, autogenous bone grafting also has a number of disadvantages, including limited availability; donor site morbidity; extension of surgical time; fast and unpredictable resorption rates, especially for extraoral donor sites such as the iliac bone; and the requirement of general anesthesia for extraoral sites [5, 6].

Deproteinized bovine bone (DBB) is a frequently used bone substitute in SFE procedures and may be used alone or in combination with another substitute [4, 7, 8]. Importantly, the osteoconductive interconnecting pore system of DBB serves as a scaffold for the migration of osteogenic cells [8–10]. Furthermore, DBB has very low substitution rates [4, 7]. Calcium sulfate is a synthetic bone graft material that has been used clinically as a resorbable material for over a century [11]. It has high biocompatibility and osteoconductivity and can be completely reabsorbed [12]. Indeed, the rapid rate of calcium sulfate resorption allows grafted areas to be replaced by newly formed bone even after just 2 weeks [11, 12].

The primary aim of the 2-stage SFE procedure is placement of implants in the grafted bone after a healing period [13]. Therefore, graft volume stability is considered a primary factor underpinning success in SFE procedures [13].

Different radiographic techniques have been reported for evaluation of grafted areas [14]. Cone beam computed tomography (CBCT) is a useful radiological method for evaluating the presence of any pathology within the sinus cavity before operation [15]. Furthermore, CBCT can be used to identify the amount of available grafted bone both before implant placement and in the period following SFE [15–17].

The aim of the present study was to use repetitive CBCT scans to compare the grafted volumes in patients receiving either DBB or a 2 : 1 mixture of novel synthetic biphasic CS and DBB as a composite. To the best of our knowledge, this is the first clinical study using computerized three-dimensional (3D) analysis and CBCT scans to compare volumetric changes in both slow resorbable bone graft substitutes and a mixture of slow and fast resorbable graft substitutes as a composite, in the augmented maxillary sinus. The null hypothesis of this retrospective study was that there would be no difference in volume reduction, after SFE, between interventions.

## 2. Materials and Methods

### 2.1. Study Design

CBCT scans of the maxillary sinuses of patients who required 2-stage (delayed implant placement) SFE augmentations were retrospectively collected from the Oral Implantology Department between February 2010 and February 2012. Only cases with repetitive CBCT images were included, specifically, cases where images were taken before the operation, within two weeks (V-I) of the operation, and six months after SFE (V-II). Patients underwent uni- or bilateral SFE with either DBB (Bio-Oss, Geistlich Pharma AG, Wolhusen, Switzerland) with a 0.25–1 mm particle size or a 2 : 1 mixture of biphasic CS (BondBone, Medical Implant System, Shlomi, Israel) with a 0.3–0.8 mm particle size and DBB (Bio-Oss, Geistlich Pharma AG, Wolhusen, Switzerland), as a composite. The inclusion criteria were as follows: repetitive CBCT history (before the operation, within two weeks (V-I) of the operation, and six months after SFE (V-II)); <5 mm of remnant alveolar portion of bone height, as evaluated by CBCT, before SFE; age > 18 years; and adequate oral hygiene. The exclusion criteria were smoking at preoperative evaluation or the healing stage (≥10 cigarettes/day), alcohol or drug abuse, maxillary sinus pathology, systemic diseases that could affect the healing process, pregnancy, uncontrolled periodontal disease, radiation and/or chemotherapy in the past, psychiatric problems, and large sinus membrane perforations that could not be repaired during SFE. The presence of any of these conditions was ascertained according to responses given in a routinely performed questionnaire that is nonspecific and administered to all SFE patients. A retrospective chart review of responses to this questionnaire, with consideration for the inclusion and exclusion criteria, yielded a final study cohort of 35 patients. This cohort was divided into 2 groups (DBB, *n* = 18; composite, *n* = 17) according to the biomaterial used, and written informed consent was obtained from all patients. The study protocol was approved by the Ethical Committee of Istanbul University, Turkey (Approval number 2015/37), and all procedures were performed in accordance with the Declaration of Helsinki.

### 2.2. Surgical Methods

**Figure 1:**
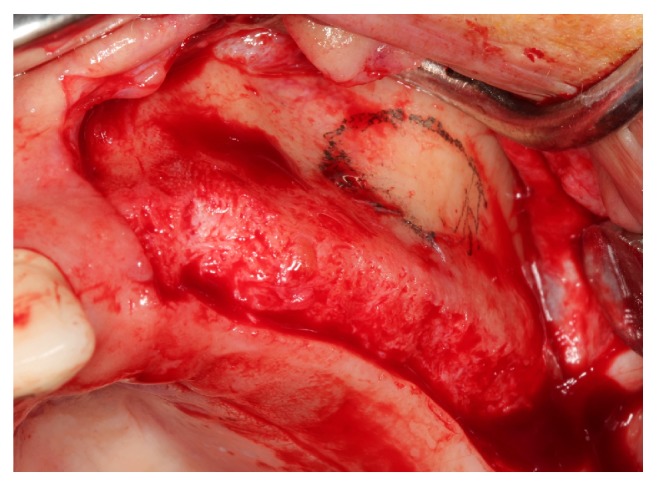
Crestal and vertical releasing buccal incisions were made along the residual alveolar bone.

**Figure 2 fig2:**
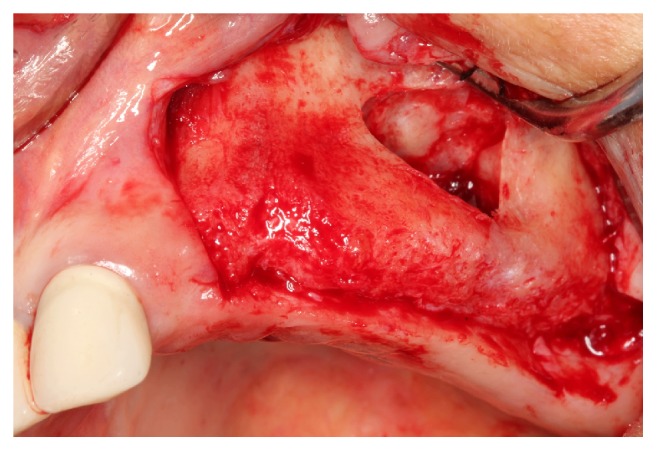
An osteotomy was performed on the buccal sinus wall using diamond rotary instruments or a piezosurgical device. The bone at the center of the access window was gently fractured, and the intact sinus membrane was gently elevated with proper instruments.

**Figure 3 fig3:**
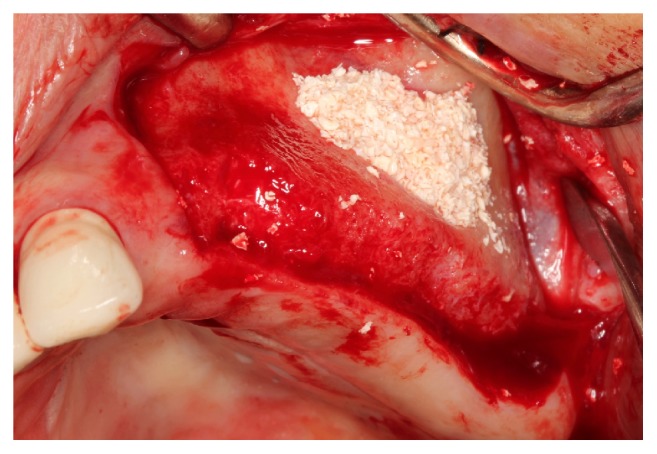
Patients requiring SFE underwent grafting procedures using either deproteinized bovine bone (DBB) or a 2 : 1 mixture of biphasic CS and DBB as a composite.

**Figure 4 fig4:**
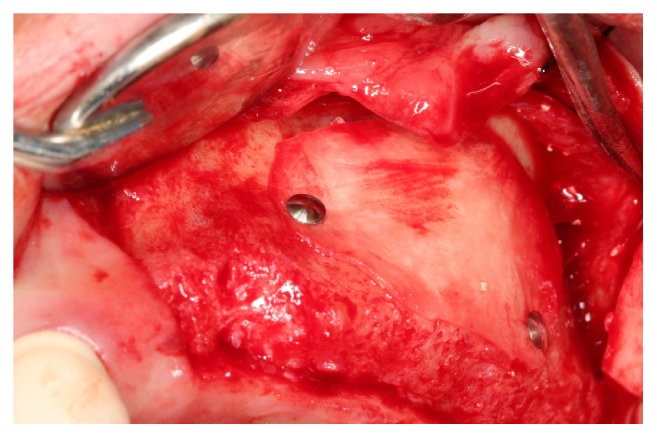
The lateral window was then covered and tacked with resorbable collagen membrane.

**Figure 5:**
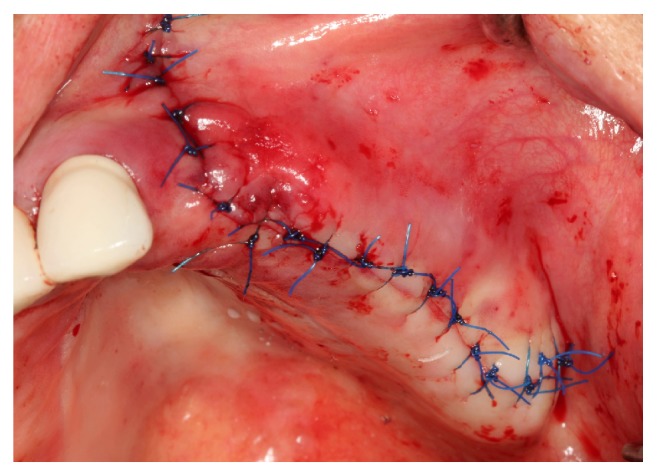
The mucosal flap was replaced with nonabsorbable monofilament material for primary closure.

Treatment was administered to patients with good periodontal health. A 2-stage approach (delayed implant placement) was used, and all surgical procedures were performed under local anesthesia (Ultracain DS Forte, Sanofi Aventis, Istanbul, Turkey). Mid-crestal and vertical releasing incisions were made along the residual alveolar bone to expose the buccal sinus wall. An oval-shaped access window was created according to the planned location of the implant and anatomy of the maxillary sinus. A mucoperiosteal flap was elevated, and the sinus membrane was accessed by drilling a window into the buccal sinus wall with a dental carbide and diamond round bur in a high-speed handpiece, under copious sterile saline irrigation. In case of thin sinus walls, piezosurgery was used for lateral window osteotomy. The bone at the center of the access window was gently fractured inward with an osteotome, and the intact sinus membrane with the remaining bone was elevated superiorly. In cases with perforation of the sinus membrane, repair was performed using a resorbable collagen membrane (Mem-Lok, Collagen Matrix, Franklin Lanes, NJ, USA). According to group allocations, the appropriate graft was gently packed until it filled the entire cavity between the sinus floor and the sinus membrane. A trimmed, resorbable collagen barrier membrane, matching the osteotomy window, was tacked (Pinfix, Sedenta, Istanbul, Turkey) onto the buccal wall of the sinus to prevent migration of the graft and soft tissue invasion. The mucosal flap was sutured using a 4-0 nonabsorbable monofilament material (SERALON, Serag-Wiessner, Naila, Germany) for primary closure (Figures 1 [Fig fig2] [Fig fig3] [Fig fig4]–5).

All patients received the same medication protocol. Postoperative care included antibiotic prophylaxis starting 1 hour before the surgery and continued for 7 days postoperatively (1000 mg amoxicillin and clavulanic acid, twice daily), pain medication (600 mg ibuprofen to be taken as needed every 6 h), and a 0.2% chlorhexidine mouthwash twice daily for 10 days from the day after the operation. Dexamethasone (4 mg orally, daily) was administered for 3 days to minimize edema. Sutures were removed 10 days after surgery. Patients were also examined for adverse outcomes, such as infection, pain, and fistula formation, during follow-up sessions at 2, 4, and 6 months after SFE. Bone grafts were left to heal for 6 months before root-form roughed surface implants were placed. These were then left for an additional 3 to 4 months to allow osseointegration before prosthetic loading. All patients received cement-retained fixed prosthetic restorations with porcelain fused to metal crowns or bridges. Clinical examination of the implants placed in the grafted sinus was conducted according to Albrektsson survival criteria, every 6 months for 2 years [18].

### 2.3. Radiographic Analysis

The intraexaminer study error was evaluated using postoperative images from 5 randomly selected SFE sites. An investigator (OB), not involved in the SFE operations, repeated the measurements; an excellent intraclass correlation coefficient of 0.972 was observed.

**Figure 6:**
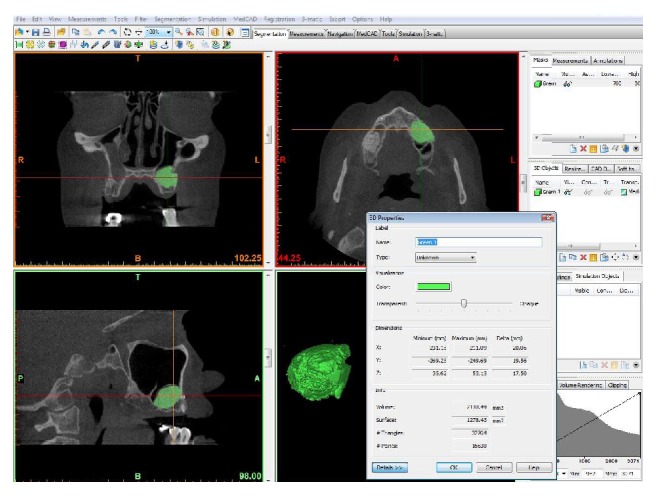
Digital reconstruction was performed by selecting the grafted volume, whereas manual reconstruction was based on threshold values selected according to the gray values of native bone, grafted bone, soft tissue, and sinus cavity, expressed on the software program.

**Figure 7:**
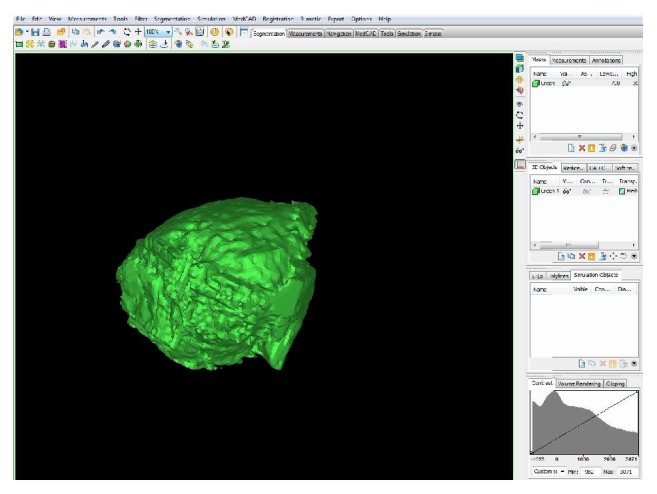
The volume of the three-dimensional grafted biomaterial was calculated.

CBCT scan data were collected in the Digital Imaging and Communications in Medicine (DICOM) file format, using the i-CAT 3D Imaging System (Imaging Sciences International Inc., Hatfield, PA, USA) with a field view of 13 × 8 cm and 0.25 voxel size. The data obtained from the CBCT images of the upper jaw were transferred to a network computer workstation, where the volumetric changes of the graft were analyzed using the MIMICS 14.0 software (Materialise Europe, World Headquarters, Leuven, Belgium). In a room with low lighting conditions, the sinus bone grafts were reconstructed in 3D to evaluate the volume changes in DBB or composite grafts at 2 reference points in time (V-I and V-II). The digital volumetric calculation methodology used has been described in previous studies [19, 20]. Digital reconstruction was accomplished by selecting the grafted volume, whereas manual reconstruction was based on threshold values selected according to the gray values of native bone, grafted bone, soft tissue, and sinus cavity, expressed in CBCT. The volume (mm^3^) of the 3D grafted object was calculated for V-I and V-II (Figures 6 and 7). The residual bone height (H-0) and width (W-0) of the alveolar ridge were measured prior to the operation at the point of planned implant insertion using software (i-CAT, Imaging Sciences International Inc., Hatfield, PA, USA). Ridge width was calculated at a level corresponding to mid-height. A single value of ridge height and width was calculated for each grafted site.

### 2.4. Statistical Analysis

Power calculation for comparison of volume reductions between the groups gave the following results: power = 0.80, *β* = 0.20, and *α* = 0.05 (in accordance with the reference related to the parameter of volume reduction of grafted sinus, Δ = 0.45, and standard deviation (SD) = 0.47). On the basis of this calculation, the necessary sample size was 11 subjects per group. Changes in grafted bone volume over time were statistically analyzed using IBM SPSS Statistics 22 (IBM SPSS, Turkey). The Kolmogorov-Smirnov test was used to assess the normality of data distribution. Student's *t*-tests were used for comparisons between groups, whereas intragroup comparisons of parameters were conducted using paired-sample *t*-tests for normally distributed data. The level of statistical significance was set at *p* < 0.05 for all analyses.

## 3. Results and Discussion

### 3.1. Results

**Figure 8:**
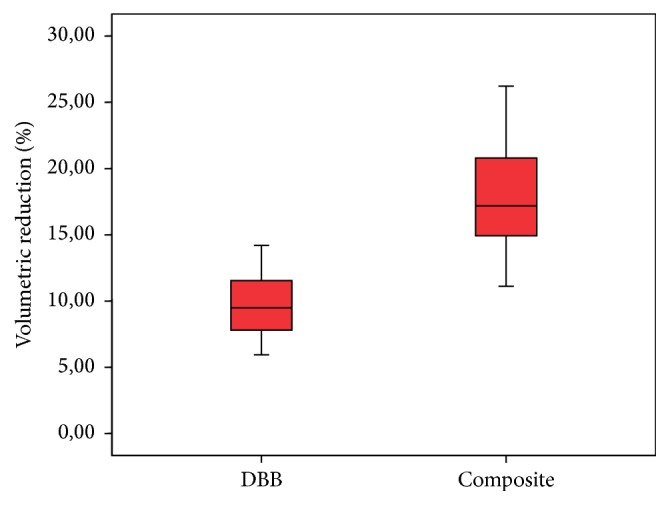
Box plot of percentages of reduction in bone graft volume in the experimental groups after 6 months. DBB, deproteinized bovine bone; composite, 2 : 1 mixture of biphasic CS and DBB.

Two of 35 patients, both belonging to the composite group, were excluded from the analysis as the border between the graft and natural sinus bone walls appeared indistinct at follow-up (V-II). In total, 33 patients with the necessary criteria, including 19 DBB and 18 composite SFE sites, were included in the study. Of these, 18 patients received DBB grafts (mean age, 52.47 ± 11.44 years) and 15 patients received composite grafts (mean age, 50.66 ± 11.65 years). Bilateral augmentation was performed in 1 case in the DBB group and in 3 cases in the composite group. Most of the sites were partial (13 DBB, 10 composite) or totally edentulous (2 DBB, 6 composite). There were 4 single tooth sites in the DBB group and 2 in the composite group. Minor perforation of the sinus membrane occurred in 2 cases of the DBB group; both of these cases were closed with the help of a collagen membrane. None of the perforations were wide enough to require abortion of SFE. No further surgical complications or adverse events were observed during follow-up. The mean residual bone height and width at the planned implant sites were 3.26 ± 0.89 mm and 7.39 ± 1.13 mm, respectively. Student's *t*-test results revealed no significant differences in the mean residual bone height and width between the DBB and composite groups (*p* > 0.05) (Table 1). Overall graft height losses of 1.72 ± 0.66 mm and 3.14 mm ±0.61 mm were observed in the DBB and composite groups, respectively, after 6 months of healing. The mean graft volume reduction rate was 13.40 ± 5.49% (1.7%–26.2%). A significant graft volume reduction was observed between V-I and V-II in both groups (*p* < 0.01) (Table 2). The average percentage volume reduction in the DBB and composite groups was 9.39 ± 3.01% and 17.65 ± 4.15%, respectively (Table 2). The DBB group exhibited significantly less volume reduction than the composite group (*p* < 0.01) (Table 2, Figure 8). A total of 73 dental implants were placed in the grafted sinuses successfully. Implant manufacturers included Biohorizons, Birmingham, AL, USA (*n* = 39), Nobel Biocare AB, Göteborg, Sweden (*n* = 21), and Medical Implant System, Shlomi, Israel (*n* = 13). No implants were lost during the 2-year follow-up period, and the survival rate was 100%, according to Albrektsson criteria.

### 3.2. Discussion

The aim of this study was to evaluate the volumetric changes associated with 2 biomaterials that had either low or high substitution rates in SFE. Selection of the ideal biomaterial is influenced by the time taken to form new bone, as well as the stability of the grafting volume [13]. Volumetric evaluation of the resorption rate of grafted bone by using three-dimensional techniques is more accurate than evaluation using two-dimensional techniques [13]. Several factors such as size of the sinus, number of missing teeth, smoking, pneumatization effect, and remaining alveolar bone may influence the amount of bone graft on maxillary sinus [3, 4, 8, 13]. Volume reduction following augmentation procedures is primarily influenced by the features of the bone grafting biomaterial [3, 4, 8]. In the present study, a significant volumetric reduction was observed in both groups; however the composite group exhibited more resorption than the DBB group. Therefore, the null hypothesis of the present study was rejected.

DBB has previously been reported as having none or limited resorption [7, 21]. Although high rates of new bone formation have been reported for DBB, the resorption capacity of the material is still of concern [13, 21]. Scarano et al. [22] observed 31% residual DBB graft material in core biopsies taken after 6 months of healing. Umanjec-Korac et al. [23] used 3D assessment to observe changes in SFE DBB grafts and noted a 19.30% volume reduction. In the literature, it has been shown that the resorption rate of DBB is between 6% and 20% prior to implant placement in 2-stage SFE operations [19, 24]. Additionally, some studies have used two-dimensional (2D) analysis to evaluate changes in grafting ridge height after SFE. Specifically, in a study by Hallman et al. [25], a reduction in linear grafting volume was observed, in addition to changes in height measured from a reference point as a representative metric of volumetric change. However, these studies may have a high risk of bias, as they evaluated volumetric changes using only linear measurements as representative of 3D alterations. Differences that exist in the volumetric reduction rates reported by different studies, even when the same graft biomaterial is used, may be explained by the anatomy of the sinus cavity, surgical method, experience of the surgeon, repneumatization force of the patient, and the technique used for measurement [9, 13, 19]. In the present study, the lowest volume reduction rate was associated with DBB, which has osteoconductive properties, structural stability, and low turnover rates [13, 19]. The very limited resorption of this material may serve as an advantage by resisting repneumatization force of the sinus, resulting in volumetric stability of the grafted region after SFE [26]. However, high-turnover resorbable biomaterials may help to fill spaces with newly formed bone during replacement, instead of residual DBB. This, in turn, increases the bone-implant contact surface and resistance to infections that may be observed in the future [4, 27].

Graft particles of CS are prone to resorption and are replaced by newly formed bone after only 1 to 4 months of healing [11, 12]. In addition, a direct source of calcium may help induce the initial stage of osteoprogenitor cell migration more rapidly [5, 14]. Therefore, in the present study, fast resorbable biphasic CS was mixed as a composite with DBB and then compared with DBB, which had very low substitution rates. The combined use of high- and low-turnover-rate materials as a composite may offer advantages. Repneumatization of the sinus, which may result in reduced overall grafting volume, can be inhibited during the healing period when using low-turnover-rate materials. In the present study, the composite group presented with a high level of graft shrinkage when compared with that of the DBB group. It is possible that the proportion of DBB grafting particles in the composite may have been insufficient to resist repneumatization forces. The grafting volume of the composite group may be more stable if the biphasic CS is used in mixture ratios less than 2 : 1. Reducing healing time may also help to decrease the detrimental effect of repneumatization. Collins et al. [28] reported that CS alone may be successfully used in the treatment of well-protected defects (e.g., socket augmentation), provided implants are placed shortly after surgery. Using high shrinkage biomaterials in SFE may result in placement of short implants after healing and also increases surgical complications during operation [9, 13].

It has been suggested that the ideal biomaterial for bone regeneration should have mechanical characteristics similar to the hard tissue to be regenerated [9, 26]. Although CS is rapidly replaced with natural bone, clinical maturation of the newly formed bone takes time, sometimes more than 4 to 6 months [29, 30]. During healing, the grafting volume of the composite may not adequately resist the ongoing pneumatization, resulting in immature characteristics of the newly formed bone. Therefore, it can be speculated that the mechanical properties of the regenerating bone should be greater, in order to maintain volume and increase resorption time, at least until placement of implants and loading.

No implants were lost during the 2-year follow-up period. According to the literature, the survival rate of implants after SFE is between 91% and 95% [3, 8, 20]. Failure rates increase when the 1-staged approach is used [3, 8, 9]. We found that a 6-month healing period was sufficient to achieve and maintain stability for 2 years in both groups with the 2-staged SFE approach.

Although the evaluation period of the present study is not very long, it has been reported that the majority of graft resorption occurs during the first 6 months of healing after augmentation [8, 14]. Therefore, with respect to stability of the grafting volume, the time period prior to implant placement is crucial for evaluation of biomaterials in 2-stage SFE procedures.

One of the primary limitations of the present study is the lack of histological and histomorphometrical analysis in the 2 groups after 6 months of healing. Although the 2-year follow-up period showed no adverse events and all implants were well integrated and loaded, we had no way of investigating the relationship between volumetric reductions and histomorphometrical measurements.

## 4. Conclusions

Within the limits of this study, it can be concluded that these bone substitutes can be successfully used alone or as a composite in SFE procedures. Significant volume reduction at the SFE site over time was observed for both biomaterials. However, DBB may offer greater volume stability over time than a composite. Reduced grafting volume did not appear to compromise the survival rates of implants in either group. A longer observation period is necessary to better understand the volumetric stability of augmented maxillary sinuses and the survival rates of implants.

## Figures and Tables

**Table 1:** Mean residual bone height and width at the implant sites prior to operation.

	Residual bone height (mm)	Residual bone width (mm)
	Mean ± SD	Mean ± SD
DBB	3.19 ± 0.96	7.54 ± 1.23
Composite	3.34 ± 0.84	7.23 ± 1.03
*p* ^1^	*0.622*	*0.408*

^1^Student's *t*-tests, *p* > 0.05.

DBB, deproteinized bovine bone; composite, 2 : 1 mixture of biphasic CS and DBB.

**Table 2:** Graft volume changes at V-I and V-II for both groups.

	DBB	Composite	*p* ^1^
	Mean ± SD	Mean ± SD	
V-I (mm^3^)	2431.12 ± 634.98	2870.34 ± 536.73	*0.030* ^*∗*^
V-II (mm^3^)	2206.89 ± 615.95	2360.06 ± 431.52	*0.389*
Volumetric change rate (%)	9.39 ± 3.01	17.65 ± 4.15	*0.001* ^*∗∗*^
*p* ^2^	*0.001* ^*∗∗*^	*0.001* ^*∗∗*^	

^1^Student's *t*-tests, ^*∗*^ *p* < 0.05 and ^*∗∗*^ *p* < 0.01.

^2^Paired *t*-tests.

DBB, deproteinized bovine bone; composite, 2 : 1 mixture of biphasic CS and DBB.
